# Co-repressor, co-activator and general transcription factor: the many faces of the Sin3 histone deacetylase (HDAC) complex

**DOI:** 10.1042/BCJ20170314

**Published:** 2018-12-14

**Authors:** Grace E. Adams, Aditya Chandru, Shaun M. Cowley

**Affiliations:** Department of Molecular and Cell Biology, University of Leicester, Leicester LE1 7RH, U.K.

**Keywords:** chromatin, deacetylase, histone, transcription

## Abstract

At face value, the Sin3 histone deacetylase (HDAC) complex appears to be a prototypical co-repressor complex, that is, a multi-protein complex recruited to chromatin by DNA bound repressor proteins to facilitate local histone deacetylation and transcriptional repression. While this is almost certainly part of its role, Sin3 stubbornly refuses to be pigeon-holed in quite this way. Genome-wide mapping studies have found that Sin3 localises predominantly to the promoters of actively transcribed genes. While Sin3 knockout studies in various species result in a combination of both up- and down-regulated genes. Furthermore, genes such as the stem cell factor, *Nanog*, are dependent on the direct association of Sin3 for active transcription to occur. Sin3 appears to have properties of a co-repressor, co-activator and general transcription factor, and has thus been termed a co-regulator complex. Through a series of unique domains, Sin3 is able to assemble HDAC1/2, chromatin adaptors and transcription factors in a series of functionally and compositionally distinct complexes to modify chromatin at both gene-specific and global levels. Unsurprisingly, therefore, Sin3/HDAC1 have been implicated in the regulation of numerous cellular processes, including mammalian development, maintenance of pluripotency, cell cycle regulation and diseases such as cancer.

## Histone acetylation: dynamic regulator of chromatin accessibility

The long and fragile genomes of eukaryotic species are packaged into histones to form the more robust chromatin fibre. Chromatin allows the efficient packaging of the genetic material but it also forms a physical barrier to RNA polymerase and transcription factors which require access to the DNA in order to initiate transcription. Therefore, to regulate access to the underlying DNA sequence, histones undergo post-translational modifications (PTM), changing their chemical properties to open or close chromatin [1]. One of the most common histone PTMs is Lysine acetylation (Lys-Ac). The unstructured N-terminal tails of core histones (H2A, H2B, H3 and H4) are rich in Lys residues, which being naturally positively charged, have an affinity for the negatively charged DNA backbone. Acetylation masks this negative charge, loosening the histone's grip around DNA and opening the chromatin to make it more transcriptionally permissible. In addition, Lys-Ac acts as a binding site for proteins bearing a bromodomain [2], which for the most part include factors that help stimulate transcription, thus reinforcing the positive nature of histone acetylation. By virtue of being a PTM of histones, the genetic packaging material, acetylation is often referred to as an ‘epigenetic’ modification. This tends to be a semantic argument, depending on your definition of epigenetics, but there are a couple points that often get overlooked in these discussions: (i) that the acetylome includes thousands of proteins in addition to histones, with dozens of roles independent of gene regulation [3,4]; and (ii) that histone acetylation is highly dynamic, with a typical half-life of ∼60–120 min [4,5], so if it does represent a code, it is a relatively short-lived one. The levels of Lys-Ac are regulated by the opposing action of histone acetyltransferases (HATs) and histone deacetylases (HDACs). There are 18 HDACs in mammals which can be categorised initially as having either Zn^2+^-dependent (Class I, II and IV) or NAD^+^-dependent (Class III — Sirtuins) catalytic domains; and then further by the presence of additional N-terminal domains and a tissue-specific expression pattern (Class II and IV) or a short C-terminal tail and ubiquitous expression (Class I) (see [6–8] for extensive reviews). Although deacetylase enzymes almost certainly have thousands of different protein substrates, and some may never enter the nucleus at all, we still tend to refer to them as HDACs because histones remain the best-understood substrate for many of these enzymes. This particular review will focus on class I HDACs, and the Sin3 complex in particular, which are targeted to chromatin by multiple mechanisms and thus among all of the deacetylase enzymes, are probably most accurately named.

## The complex world of HDAC1/2 function

The highly related deacetylases, HDAC1 and HDAC2 (HDAC1/2) share 82% amino acid identity and form the catalytic core of numerous co-repressor complexes, which account for ∼50% of all cellular deacetylase activity in embryonic stem (ES) cells [9] and T-cells [10]. The four canonical HDAC1/2 complexes are Sin3, NuRD, CoREST and MiDAC [6,11–13]. These multi-protein complexes are critical to the function of HDAC1/2. HDAC1/2 have reduced activity in the absence of a binding partner [14,15], and they are effectively blind, requiring complex components to mediate the essential protein–protein interactions which guide them to substrates. But why so many? Indeed, it is difficult to think of another major enzyme that is part of four distinct biochemical entities. The specificity of protein substrates, target genes and the combination of enzymatic activities (e.g. deacetylase/demethylase) may go some way to explaining the superabundance of HDAC1/2 complexes. It is essential that the deacetylation machinery is not only tightly regulated, but highly specific. To date, we have little understanding as to how this specificity is achieved, although it appears that it is driven by the holistic action of the fully assembled complexes. We hypothesise that unique subunit within each complex help ‘present’ different protein targets to the HDAC1/2 catalytic site, through a combination of protein–protein interactions, and/or histone recognition. Understanding the assembly of different subunits, within the holo-complex, is therefore critical to understanding the mechanism of its substrate recognition *in vivo*. The complex may also play a role in the regulation of HDAC1/2 activity through the recognition of inositol phosphates (InsP). The ELM2-SANT domain of MTA1 (part of the NuRD complex) contains specific and highly conserved basic residues which help co-ordinate the negatively charged phosphates in InsP6 [16]. These residues, and the stimulation of the purified complex via InsP levels, appears to be conserved in the NuRD and MiDAC complexes through their HDAC1/2 interacting subunits, MTA1 and MIDEAS, respectively [13]. The Sin3 complex conspicuously lacks an ELM2-SANT domain and appears to be unique amongst HDAC1/2 complexes by being insensitive to InsP *in vitro* [17]. Nonetheless, Sin3/HDAC1 remains a critical regulator of gene expression and as discussed in detail below, is essential for embryo development and the differentiation of numerous tissue types.

## The Sin3 co-regulator complex(es)

In many regards, Sin3 is the prototypical co-repressor complex, that is, a multi-protein complex recruited to chromatin by DNA bound repressor proteins to facilitate local histone deacetylation and transcriptional repression [18–23]. While this is almost certainly part of its role, Sin3 stubbornly refuses to be pigeon-holed in quite this way. Loss of Sin3 in yeast, fruit flies and mice results in a combination of up- and down-regulated genes, indicating roles in both transcriptional activation and repression [24–27]. Furthermore, genome-wide chromatin immunoprecipitation (ChIP) studies also show Sin3 predominantly bound within the vicinity of transcriptional start sites, not repressed loci [27,28]. HDAC1 activity [29] and the Sin3A complex [30] are also required for full transcriptional activity of interferon-α responsive genes and Nanog promoter, respectively. Sin3 appears to have properties of a co-repressor, co-activator and general transcription factor, and has thus been termed a co-regulator complex [31].

**Figure 1.**
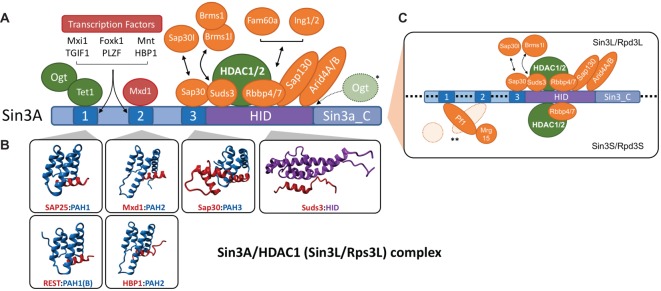
Schematic of Sin3A/HDAC1 complex. (**A**) Numbers indicate PAH domains 1–3. HID, HDAC-interaction domain; Sin3a_C, Sin3A C-terminal domain. Transcription factor (red) binding to Sin3A occurs predominantly via PAH1 and 2 as indicated. Chromatin-associated proteins are coloured orange and enzymes in green. (**B**) NMR structures of isolated PAH domains bound to the SID of the indicated factor. PAH domains are shown in blue, SIDs in red and HID in purple. All data were taken from the Protein Data Bank (PDB code indicated in brackets), SAP25:PAH1 (2RMS), REST:PAH1 (Sin3B, 2CZY), Mxd1:PAH2 (1G1E), HBP1:PAH2 (1S5R), Sap30:PAH3 (2LD7) and Suds3:HID (2N2H). (**C**) Sin3 may be subdivided into two major complexes — Sin3L/Rpd3L (L — large) and Sin3S/Rpd3S (small). *In vivo*, the Sin3A complex forms the scaffold of the larger Sin3L/Rpd3L complex, while Sin3B fulfils the same role in the Sin3S/Rpd3S complex. * OGT binds to both Tet1 and Sin3A. ** Pf1 has two SID domains. Pf1 SID2 (PHD2) binds to PAH1, while SID1 (PHD1) can interact with both MRG15 and PAH2 in a manner that is mutually exclusive. *** Arid4A (RBP1) can associate with Sin3 via Sap30 [85], while binding of Arid4B (Sap180) was mapped to the HID [38].

The central platform of the complex is the Sin3 protein, which binds directly to HDAC1/2 and many other adaptors [18,21,22], to assemble a functional, enzymatically active, Sin3 complex (Figure 1). Mammals contain two highly related Sin3 proteins, Sin3A and Sin3B [32,33] which share an overall domain structure and an association with HDAC1/2. However, as will be discussed in detail below, mouse knockout (KO) studies have shown them to be non-redundant and there is evidence that they form distinct protein complexes. Both proteins are able to integrate signalling and transcription regulation by interacting with a host of transcription factors (REST, Mxd1, PLZF, HBP1, etc.) which bind specifically to one of three paired-amphipathic helix (PAH) domains (Figure 1 and [32,34–36]). They are also recruited to chromatin through a series of Sin3-associated proteins (SAP). These include, AT-rich interaction domain (ARID), tudor and chromodomain within Arid4A/B, a PHD finger in Ing1/2 which bind directly to methylated Lys within histone tails and the histone chaperones, Rbbp4/7 [22,37–39]. In addition to deacetylase activity, Sin3A is also associated with DNA hydroxylases, Tet1 and Tet3 [28,40] and the *O*-GlcNAc transferase, OGT [41]; although, how these activities synergise with deacetylase activity is not fully understood. An extensive catalogue of SAPs can be found in the following studies [11,40,42–44].

Understanding how different SAPs co-operate to recruit the Sin3 complex to chromatin is a crucial step towards understanding its functions in cells. With a dozen or more different SAPs it raises the question: are they all bound at the same time? And if not, how many different variants of the Sin3 complex are there? To address these questions Streubel et al. [43] employed two independent co-immunoprecipitation experiments (with different antibodies), coupled to quantitative mass spectrometry, to assess the stoichiometry of individual complex components. Intriguingly, given the panoply of available factors, it suggests the core-Sin3A complex consists of Sin3A, Sap30, Rbbp4/7 and HDAC1 (preferentially over HDAC2). The notion that Sin3A may have a preference for HDAC1 over HDAC2 is supported by data from T-cells, where a Sin3A/HDAC2 association can only be detected following deletion of HDAC1 [10]. Also of note is the sub-stoichiometric nature of Suds3, an essential gene thought to enhance the association of Sin3 with HDAC1 [45,46], which we might have predicted to be closer to a 1 : 1 ratio with Sin3A. Suds3 may be interchangeable with the related factors, Brms1/Brms1l [47,48], and that cumulatively all three proteins aid association with HDAC1 [49]. The majority of SAPs (Tet1, OGT, SAP25, etc.) and transcription factors (Mxi1, Foxk1, etc.) have a relative stoichiometry of <0.1 compared with Sin3A and therefore occupy only a fraction of the total Sin3A in cells, arguing that competition for binding sites (e.g. Sap25 [50] and REST [51] for PAH1) may not be as prevalent as first imagined. If so, then multiple varieties of Sin3A complexes may exist contemporaneously in cells. Cell type may also dictate complex composition, as the association of Fam60a, Tet1 and OGT all appear to be specific to ES cells, rather than somatic cells [43]. The definition of a Sin3A complex may therefore be a moveable feast, consisting of constitutive factors (HDAC1, Sap30, etc.) and a variety of sub-stoichiometric proteins and transcriptional factors that are assembled dependent on the cell type.

One well-studied sub-division among Sin3 complexes comes from work in yeast. Purification of Sin3/Rpd3(HDAC1) complexes identified two distinct biochemical entities of different apparent molecular mass, termed Rpd3 large (Rpd3L: Sin3, Rpd3, Sap30, Sds3, Ume1, Ume6 and six other proteins) and Rpd3 small (Rpd3s: Sin3, Rpd3, Ume1, Rco1 and Eaf3) [52,53]. Rpd3L seems to perform the classical co-repressor role being recruited to gene-specific loci by transcription factors such as Ume6. While Rpd3S is recruited in the wake of active RNA polymerase II, via the association of H3 Lys36 tri-methylation (H3K36me3) and the chromodomain of Eaf3, to repress cryptic promoters in actively transcribed regions. Loss of Eaf3 or Set2, the methyltransferase responsible for depositing H3K36me3, resulted in the activation cryptic promoters and the synthesis of spurious transcripts [52]. The mammalian equivalent of Eaf3, Mrg15, was found to co-purify with Sin3B, HDAC1 and Pf1 (Rco1s), but significantly, not with Sin3A. Biochemical data from many studies suggests that Sin3A may function as Sin3L/Rpd3L and Sin3B as Sin3S/Rpd3S. To cement this idea it is useful to compare and contrast many complex purification studies. Two different studies that utilised co-IP and mass spectrometry of endogenous Sin3A complexes [40,43] failed to detect either Pf1 or Mrg15, which suggests they are not major components of Sin3A complexes. While conversely, Nishibuchi et al. [54] performed Flag-Mrg15 co-IP experiments in HeLa cells and were able to pulldown Sin3B, Pf1, Mrg15, HDAC1/2, KDM5A and EMSY, but did not detect Sin3A, Sap30 or Suds3. Jelinic et al. [55] were able to reconstitute a mammalian tetrameric complex equivalent to Rpd3S using Sin3B, HDAC1, Mrg15 (Eaf3) and Pf1 (Rco1), which they refer to as the SHMP complex. Mrg15 recruitment into this complex requires Pf1, which in turn does not bind to Sin3A, thereby establishing a binary distinction between the two complexes. Although Sin3A appears to form no part of the Sin3S/Rpd3S complex, there is evidence to suggest that Sin3B may still form part of a Sin3L/Rpd3L complex [56]. Intriguingly, Mrg15 and Eaf3 seem to act as double agents, being present in both HDAC and HAT complexes [52,57,58], suggesting that the recognition of H3K36me3 could result in either histone acetylation or deacetylation read-outs.

## Sin3 domain structure: lessons from structural biology

The domain structure of Sin3A/B is highly conserved from yeast to man [32]. From N- to C-terminus, it contains, three PAH domains (Pfam: PF02671), an HDAC-interaction domain (HID, Pfam: PF08295) and a Sin3A C-terminal domain (Sin3a_C, Pfam: PF16879), formerly referred to as the highly conserved region (HCR [59,60]), an eccentric term since much of the protein is highly conserved. Sin3a_C contains the region which previously included PAH4, a non-canonical PAH domain lacking critical protein–protein interacting residues [61]. Akin to the reclassification of Pluto [62], the outermost PAH domain no longer fulfils the criteria required of its inner neighbours. Both Sin3A and Sin3B share this modular arrangement of PAH1-3, HID and Sin3a_C. While the two former domains have well-defined roles discussed in detail below, the Sin3a_C region (887–1190 aa) is a little more enigmatic. The binding site for OGT was mapped to Sin3A residues 888–967 [41], but OGT may also be recruited to the complex via Tet1 and its association with PAH1 [63]. Structural biology has proved to be an extremely powerful tool in understanding the molecular details of these Sin3 domains.

## PAH domains: under lock-and-key

The three PAH domains are imperfect ∼100 amino acid repeats with a conserved fold, consisting of four α-helices separated into pairs by a central loop [35,51,61,64–66] (Figure 1). These amphipathic helices arrange themselves to form a hydrophobic cleft into which the single helix Sin3-interaction domain (SID) of the interacting partner is able to insert and bind with high affinity. The PAH domains act as critical protein–protein docking sites, permitting the formation of Sin3 complexes by allowing multiple transcription factors and chromatin-associated factors to recruit the core deacetylase activity [19,32,43,56,67]. The first structural studies of a PAH:SID interaction were performed with proteins of the Mxd1 family, the original baits used in two-hybrid screens to isolate mammalian Sin3 [32,33], bound to PAH2 of Sin3A [61] and Sin3B [65]. These revealed a highly conserved set of hydrophobic interactions, with critical residues in helix1 (Ile308/Val311) and helix2 (Leu329/Leu332) of Sin3A-PAH2 accommodating those of the Mxd1-SID (Leu12/Ala15/Ala16) [68]. An analogous set of interactions occurs between helices 1 and 2 of PAH1 with the SIDs of REST [51] and Sap25 [64] (Figure 1). One significant difference between the two domains is that Sin3A-PAH1 is largely structured in the absence of a SID, whereas Sin3A-PAH2 is not; the latter undergoing a mutual folding transition with its ligand upon binding. Interestingly, the analogous folding transition in Sin3B-PAH2 is limited to residues in helix1 [65]. Although PAH1 and PAH2 are thought to function as independent domains [69], there is also some evidence for cooperativity, since mutations in PAH1 can affect binding of the Mxd1-SID to PAH2 [68], despite not binding to PAH1. Unlike PAH1 and 2, which engage in sub-stoichiometric interactions with a range of transcription factors and chromatin-associated proteins, PAH3 (of Sin3A) is constitutively bound to Sap30 [43]. The PAH3/Sap30 structure has many unique features including a tri-partite Sap30-SID, which interacts with both the canonical hydrophobic cleft and an additional hydrophobic surface on the side of PAH3 [70]. Consistent with the constitutive nature of their association, PAH3/Sap30 have the highest affinity of any PAH/SID combination (∼10 nM) thus far measured.

All three PAH domains show a significant degree of similarity and yet the specificity of interacting partners is quite distinct. The Mxd1-SID binds PAH2 but does not interact with PAH1 or 3 [64,68]; while the SAP25-SID binds to PAH1 but not PAH2 or 3 [50,64]. This specificity is derived from the unique arrangement of long and short hydrophobic side-chains which from a unique lock-and-key interaction for each combination of PAH:SID. These high-affinity hydrophobic interactions (typical *K*_d_ in the sub-micromolar region, 50–200 nM) are guided by long-range electrostatic interactions. van Ingen et al. [71] observed that addition of a four amino acid sequence (Arg/Arg/Glu/Arg) to the Mxd1-SID improved the *K*_d_ from 1.4 µM (Mxd1 5–20aa) to 0.4 µM (Mxd1 5–24aa). The increased affinity was the result of a long-range electrostatic attraction between Sin3B Lys165 (which sits above the hydrophobic cleft) and Mxd1 Glu23. An N:O bridge forms through hydrogen bonding which orients the SID for binding and brings it close enough to the PAH domain for the relatively short range hydrophobic interaction to occur. Sin3A-PAH2 contains an equivalent Lys residue, Lys315, whose charge also contributes to the binding of the Mxd1-SID [61], suggesting a conserved manner of recruitment to Sin3A and Sin3B in this instance. Electrostatic interactions also contribute to the association of PAH1, PAH3 and HID with their binding partners [49,64,70].

In closing this section, it may be useful to reflect on the usage of the PAH domains. Data from Streubel et al. show that Sap30 is present in a 1 : 1 complex with Sin3A suggesting that PAH3 may be permanently occupied [43]. This would leave the majority of the burden (numerically at least) on PAH1 and PAH2 to mediate binding with upwards of 20 different interacting partners. If the aim were to inhibit the Sin3A complex therapeutically, as an alternative method of HDAC inhibition in cells, then this would be a logical place to start. Indeed, many studies from Waxman and colleagues have shown that selective inhibition of PAH2 with an interfering peptide derived from the Mxd1-SID termed, SID-decoy, reduces the growth of triple negative breast cancer (TNBC) cells [72]. Interference with Sin3 function induces epigenetic reprogramming and differentiation in breast cancer cells through de-repression of E-cadherin, oestrogen receptor alpha (ERα) and retinoic acid receptor alpha (RARα). An *in silico* screen of small molecule inhibitor mimetics identified FDA-approved avermectin derivatives as PAH2 binders [73] which phenocopied the SID-decoy and, in conjunction with a RARα agonist, prevented metastases and improved survival following tumour removal in TNBC model mice — highlighting the potential therapeutic role of Sin3 inhibitors in cancer treatment [74].

## The HID: the enzymatic core

The binding site for HDAC1 in Sin3A was initially mapped between PAH3 and PAH4 (still a domain in 1997) and duly named the HID [21]. This is a HCR in Sin3A/B and across different species, the size of a moderate protein (∼300 aa) which mediates interactions with the aforementioned HDAC1, as well as HDAC2, Suds3, Brms1, Brmsl1, MRG15, Sap130, Arid4B (Sap180) and Rbbp4/7 [38,47,48,75,76]. Although some of these associations are clearly direct (e.g. Suds3:HID) [49], others may be indirect, MRG15 requires the presence of Pf1 to bind to Sin3B for instance [55]. The presumptive role for this gaggle of associations is to stabilise the Sin3/HDAC1 interaction. And in addition, recruit the complex to chromatin via MRG15 (Chromodomain) [77], Arid4A/B (ARID, Tudor and Chromodomain) [38] and the histone chaperones, Rbbp4/7 (WD40 domain) [78]. This latter role may occur based on the presence of the appropriate histone, or histone modification and represents a method of Sin3A/HDAC1 recruitment independent of the transcription factor:PAH domain interactions described above. As already discussed, Suds3, Brms1 and Brms1l are paralogues that share structural and sequence homology (SDS3-like, Pfam: PF08598) including two coiled-coil regions and a C-terminal SID [49], suggesting that their roles may be interchangeable. In *Saccharomyces cerevisiae*, which contains a single Suds3 gene, deletion causes dissociation of Sin3 and HDAC1 (Rpd3), and the remaining HDAC1 is enzymatically inactive [46]. Deletion of Suds3 is embryonic lethal in mice and produces defects in pericentric heterochromatin in fibroblasts [45], indicating that it has non-redundant roles with Brms1/Brms1l. The presence of a coiled-coil domain in all three proteins indicates that the Sin3A complex is likely to be a dimer; indeed, Suds3 and BRMS1 are able to form both homo- and heterodimers [49]. Dimerisation is a common feature of HDAC1/2 complexes [13], which may reflect the presence of two N-terminal tails for each core histone within the nucleosome.

A solution structure of the Suds3-SID bound to a portion of the HID (Sin3A 601–742 aa) has been described with a sub-micromolar affinity [49]. Characteristic of other Sin3:SAP interactions, it consists of a multi-helical Sin3 domain bound to an extended single helix domain of the partner. In this instance, the HID forms a six-helix bundle in which the two longest helices (α1 and α5) form an intramolecular coiled-coil stalk, that the shorter helices (α2, α3 and α4) pack against to form a globular head (Figure 1). The Suds3-SID (201–234 aa) consists of an extended N-terminal segment immediately followed by a 13-residue helix, with the latter making extensive hydrophobic and a few key electrostatic contacts with the globular head of the HID. Intriguingly, Clark et al. [49] found that recruitment of HDAC1 to the Sin3A-HID was independent of Suds3, an unexpected result given previous data from yeast [46]. However, given the close proximity of their binding sites within the HID it still seems likely that there may be some complementary, although non-essential, contacts between Suds3 and HDAC1. Structural information of a Sin3/Suds3/HDAC1 ternary complex would surely answer many of these molecular details and is eagerly anticipated.

## The physiological roles of mammalian Sin3 complexes

**Figure 2.**
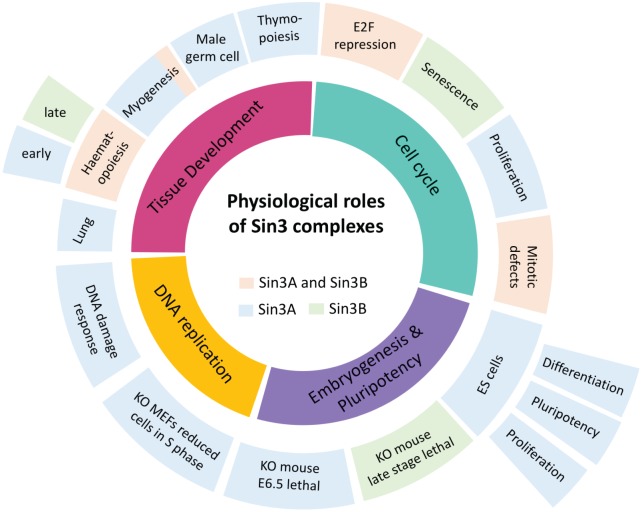
Representation of the physiological roles of Sin3 complexes in mammalian cells and tissue development. Inner ring represents the cellular process, with the outer rings denoting the specific complex functions. Processes in which Sin3A (blue), Sin3B (green) or both (orange) have been implicated are indicated.

The Sin3/HDAC complex is abundant, stable and ubiquitously expressed in mammalian tissues and cells. The temporal and cell type-specific nature of its activity, in numerous cellular processes, is mediated via transient association with transcription factors. Sin3 can therefore be viewed as a permanent tool in the chromatin toolbox, which can be recruited to specific loci and then applied to regulate chromatin accessibility in particular pathways by tissue-specific factors which interact with PAH, HID or Sin3a_C domains. Since its initial discovery as a transcriptional repressor of Myc target genes, via the association of PAH2 and Mxd1, Sin3 has been associated with numerous cellular processes, including mammalian development, maintenance of pluripotency, cell survival, cell cycle regulation (summarised in Figure 2) and diseases such as cancer.

## Distinct roles for Sin3A and Sin3B in cell cycle and DNA replication

The cell cycle is regulated at every stage (G1/S restriction point, DNA replication, G2/M check-point, mitosis) and Sin3 appears to play a role in each of them. The implication that Sin3 had a role to play in cell cycle was clear from the outset. Mxd1 (formerly Mad1) and Mxi1 are repressor proteins which compete with Myc for their common heterodimeric partner, Max, and form an anti-proliferative counter-weight to the pro-growth role of Myc [79]. Yeast two-hybrid screens performed with Mxd1/Mxi1 identified mammalian Sin3A and Sin3B as a cognate co-repressor; with later studies confirming that interaction with Sin3 (via PAH2) was required for both repression and regulation of cell cycle [32,33,80]. Latterly, Sin3 was found to be associated with many cell cycle regulators, including the master regulator of G1/S transition, retinoblastoma protein (Rb). Rb can itself be viewed as a co-repressor protein, which sits atop the E2F family of transcription factors, negatively regulating target genes preventing entry into S-phase [81]. Rb was initially demonstrated to perform this role by recruiting HDAC1 [82–84] and latterly this was shown to be as part of the Sin3A/HDAC1 complex, recruited via Arid4A (previously RBP1) and Sap30 [85]. The association of Sin3A/HDAC1 with Rb and the Mxd1 family suggests that loss of Sin3A would cause cells to cycle uncontrollably, but in fact the opposite is the case. Sin3A-KO studies in mouse embryo fibroblasts (MEFs), ES and T-cells have shown that a reduction in Sin3A levels correlates with a loss of proliferative potential [25,40,86]. This is perhaps not wholly surprising as the treatment of cells with HDAC inhibitors, such as SAHA or MS-275 (which specifically target class I HDACs), universally results in a loss of cell growth [87]. Similarly, double deletion of HDAC1/2 in MEFs causes an arrest in G1 due to the up-regulation of the CDK inhibitors, p21 and p57 [88,89]; up-regulation of p21 is also observed in Sin3A-KO MEFs [25]. Despite a loss of cell proliferation, Sin3B protein levels are unaltered in Sin3A-KO MEFs confirming distinct roles in cell cycle [25,86,90]. Although Sin3B-KO MEFs reveal no cell cycle defects, they fail to exit the cell cycle upon loss of serum in a similar manner to Rb null MEFs [91], suggesting Sin3B may be regulating cell cycle exit [92]. Moreover, loss of Sin3B in MEFs results in the absence of senescence upon oncogenic stress, while overexpression of Sin3B promotes cell cycle exit [93]. Sin3B/HDAC1 are co-localised with E2F4, p107, p130 at target genes such as cyclin A and E2F1 in quiescent cells to prevent re-entry into cell cycle [94].

Replication of the genome is an essential and rate-limiting phase of cell cycle. Critically, new DNA requires new chromatin. Histone H4 is initially deposited on nascent DNA in an acetylated form (H4K5ac/H4K12ac [95,96]) which must be deacetylated before the pattern of pre-replication PTMs can be reapplied. iPOND (isolation of proteins on nascent DNA), a technique which utilises EdU incorporation into DNA to label and purify proteins close to replisomes, identified HDAC1/2 and 3 as being present close to the replication fork [97]. Consistent with this data, loss of HDAC1/2 causes a reduction in the rate of DNA replication/fork velocity and a propensity for defects in fork progression [98]. Although these studies do not implicate the Sin3A complex directly, it seems a likely candidate among the stable of HDAC1/2 complexes. Tantalisingly, Dannenberg et al., observed a reduction in S-phase cells in Sin3A-KO MEFs, with a distinct subset of cells which had no BrdU incorporation [25]. An accumulation of errors during S-phase may contribute to the increase in Sin3A-KO cells in G2/M. KO of the Sin3A component, Suds3, also causes G2/M arrest and profound aneuploidy due to a perturbation in chromosome segregation [45]. This is reminiscent of Sin3-KOs in *Schizosaccharomyces pombe* [99], with both the mammalian and yeast phenotypes thought to occur due to the aberrant acetylation of pericentric heterochromatin, which ultimately causes the mitotic defects. A similar G2/M arrest is found in ES cells lacking Sin3A, with the subsequent triggering of the DNA damage response [40]. In summary, Sin3 complexes combine regulation of individual target genes (Rb-E2F axis, Myc/Mxd network, etc.) with house-keeping roles as a global chromatin rheostat (DNA replication, peri-centric heterochromatin, etc.) in the progression of (or exit from) cell cycle.

## Sin3 complexes are essential for embryogenesis and stem cell pluripotency

Although Sin3A and Sin3B are 57% identical [32] and share a conserved domain structure they have unique functions during development. Sin3B-KO mice show lethality during the later stages of embryonic development and display defects in erythrocytes and bone differentiation beyond embryonic day (E)14.5 [92]. These defects in terminal differentiation can be linked to the role of Sin3B/HDAC1 in cell cycle exit [90]. In contrast, Sin3A-KO embryos are not found after E6.5 [25,86]. Cultured Sin3A-KO blastocysts (E3.5) have a reduced proliferative potential suggesting that either cell cycle or pluripotency is impaired [86]. However, most Sin3A null embryos still undergo implantation, but by E5.5 few embryos were detected and these had completely lost their embryonic compartment [40]. The different phenotypes displayed by Sin3A and Sin3B-KO models reveals a lack of redundancy, in agreement with the unique biochemical and cell cycle activities discussed above.

Consistent with early embryonic lethality in mice, Sin3A has been reported to be crucial for the maintenance of pluripotency in ES cells [30,40,42,43,100,101]. Indeed, Sin3A levels are conspicuously high in ES cells, with a reduction occurring following differentiation [42,101]. A reduction in Sin3A levels also caused an extended G1-phase in ES cells [43,101], which is a critical determinant of self-renewal versus lineage commitment. The absence of Sin3A, or HDAC1/2, resulted in a down-regulation of the key pluripotent regulator, Nanog [30]. ChIP experiments showed that Sin3A binds directly to a Nanog enhancer suggesting it is required for transcriptional activity for key elements of the pluripotent network of transcription factors. In addition to maintaining pluripotency, Nanog and the Sin3A complex are able to associate and promote the reprogramming of somatic cells into induced pluripotent cells [42]. Streubel et al., identified an ES cells specific Sin3A complex which includes Tet1, OGT and Fam60a [43]. Loss of Fam60a caused a significant reduction in the recruitment of Sin3A to target genes identifying this cofactor as a critical determinant for target gene recruitment. Unsurprisingly therefore, knockdown of either Sin3A or Fam60a caused a reduction in cell proliferation and pluripotency. The 5-methyl cytosine (5mC) hydroxylase, Tet1, while not essential for the maintenance of pluripotency itself, is recruited to Sin3A via PAH1 [63,101] and appears to co-operate in the regulation of key ES cell signalling pathways [101]. Sin3A helps recruit Tet1 to genes such as the Nodal antagonist, Lefty1, maintaining its transcriptional activity and thus preventing commitment towards a mesendodermal lineage. However, ectopic expression Lefty1 alone is not able to rescue the Sin3A knockdown phenotype indicating that there are additional targets of the Sin3A/Tet1 partnership.

## Sin3 in tissue development

Sin3A and Sin3B are highly expressed in all tissues (although absolute levels may vary a little). As permanent members of the chromatin toolbox, they can be recruited by a wide variety of tissue-specific transcription factors during tissue development. It is therefore unsurprising that KO studies have identified key roles for both proteins in a variety of tissue types. Sin3A/B are critical for the maintenance of haematopoietic stem cell homeostasis, with the different isoforms of Sin3 potentially regulating alternative pathways via unique interactions with transcription factors. Loss of Sin3A in the bone marrow of mice results in a reduction in haematopoietic stem cells and subsequent lineages indicating a defect in early haematopoiesis [102]. Deletion of Sin3B in the haematopoietic lineage in mice led to elevated numbers of multipotent progenitors of haematopoietic stem cells with defects in their terminal differentiation potential, but had no effect on cell viability [90]. Sin3A has also been implicated in T-cell development. Deletion of Sin3A in early T-cells using Lck-Cre caused a 3-fold increase in double negative (DN) T-cells and a concomitant reduction in cellularity, indicating a block in thymopoiesis [86]. Conditional deletion of Sin3A in the myotube using Myf5-Cre results in perinatal lethality 24 h after birth, while the analogous Sin3B-KO mice survive for up to 2 years, indicating distinct effects on muscle differentiation [103]. Moreover, MCK-Cre driven loss of Sin3A in skeletal progenitors results in lethality at 12 days due to disorganised sarcomeres, while Sin3B-KO mice are viable. Interestingly a double KO of Sin3A and Sin3B results in an enhanced phenotype suggesting partial redundancy [103]. Analysis of muscle development genes and adhesion complexes revealed reduced expression upon deletion of Sin3A or Sin3A/B, but not Sin3B alone, suggesting Sin3A may be acting to enable their expression and maintain sarcomere formation. Sin3A has also been implicated in male germ cell development as conditional KOs (Vasa-Cre) results in sterile male mice with a sertoli only phenotype at day 10 postnatal (lacking germ cells, as marked by TRA98 and GCNA1 staining) [104]. Interestingly, analysis of postnatal pups at day 1–3 reveals germ cells are present in the testes suggesting that Sin3A is not needed for differentiation but for maintenance of germ cell viability. This is further supported by enhanced caspase-3 expression and DNA damage in germ cells as they re-enter mitosis after birth [104]. The authors linked the phenotype to up-regulation of c-Myc genes due to reduced Mxd-family repression via Sin3A and enhanced DNA damage. Finally, Sin3A plays a critical role during lung development, as loss of activity in the early foregut endoderm of the developing mouse leads to widespread defects and neonatal death [105]. Defects included down-regulation of endodermal genes and induction of a senescent-like state, consistent with up-regulation of cell cycle inhibitors p16 and p21, further supporting the role of Sin3A in cell cycle regulation.

## Future perspectives

As both global chromatin regulator and gene-specific transcriptional co-regulator, the Sin3 complex, or rather complexes, play roles in all nuclear processes. The flexibility of multiple direct protein–protein interfaces (PAH, HID and Sin3a_C domains) and a multitude of cofactors (Tet1, OGT, Arid4A, Ing2, etc.) allow its recruitment for the regulation of chromatin homeostasis throughout the cell cycle. Any process which requires access to DNA (transcription, DNA replication and/or repair) will require the manipulation of histone acetylation and as a major HDAC complex, Sin3 will likely play a role. Understanding the complex set of protein–protein interactions, and perhaps teasing these apart as a targeted therapy, will drive Sin3 biology for many years to come.

## Abbreviations

ARID: AT-rich interaction domain
ChIP: chromatin immunoprecipitation
DN: double negative
ES: embryonic stem
HATs: histone acetyltransferases
HCR: highly conserved region
HDACs: histone deacetylases
HDAC1/2: histone deacetylase 1 and 2
HID: HDAC-interaction domain
KO: knockout
Lys-Ac: lysine acetylation
MEFs: mouse embryo fibroblasts
OGT: *O*-GlcNAc transferase
PAH: paired-amphipathic helix
PTM: post-translational modifications
RARα: retinoic acid receptor alpha
SAP: Sin3-associated proteins
SID: Sin3-interaction domain
TNBC: triple negative breast cancer
